# Use of PCR in the diagnosis of pericardial amebiasis: a case report and systematic review of the literature

**DOI:** 10.1186/s12879-021-06590-x

**Published:** 2021-09-16

**Authors:** Takahiro Matsuo, Akira Saito, Fujimi Kawai, Kazuhiro Ishikawa, Ryo Hasegawa, Takahiro Suzuki, Takahisa Fujino, Katsuhito Kinoshita, Taku Asano, Atsushi Mizuno, Kenji Yagita, Nobuyuki Komiyama, Yuki Uehara, Nobuyoshi Mori

**Affiliations:** 1 Department of Infectious Diseases, St. Luke’s International Hospital, 9-1, Akashi-cho, Chuo-ku, Tokyo, Japan; 2 Department of Cardiovascular Medicine, St. Luke’s International Hospital, Tokyo, Japan; 3 St. Luke’s International University Library, Tokyo, Japan; 4 Department of Internal Medicine, St. Luke’s International Hospital, Tokyo, Japan; 5 Department of Parasitology, National Institute of Infectious Diseases, Tokyo, Japan; 6 Department of Clinical and Laboratory, St. Luke’s International Hospital, Tokyo, Japan

**Keywords:** Amebiasis, Pericarditis, *Entamoeba histolytica*, Polymerase chain reaction

## Abstract

**Background:**

*Entamoeba histolytica* (*E. histolytica*) is rarely identified as a cause of amebic pericarditis. We report a case of amebic pericarditis complicated by cardiac tamponade, in which the diagnosis was missed initially and was made retrospectively by polymerase chain reaction (PCR) testing of a stored sample of pericardial fluid. Furthermore, we performed a systematic review of the literature on amebic pericarditis.

**Case presentation:**

A 71-year-old Japanese man who had a history of sexual intercourse with several commercial sex workers 4 months previously, presented to our hospital with left chest pain and cough. He was admitted on suspicion of pericarditis. On hospital day 7, he developed cardiac tamponade requiring urgent pericardiocentesis. The patient’s symptoms temporarily improved, but 1 month later, he returned with fever and abdominal pain, and multiple liver lesions were found in the right lobe. Polymerase chain reaction of the aspiration fluid of the liver lesion and pericardial and pleural fluid stored from the previous hospitalization were all positive for *E. histolytica*. Together with the positive serum antibody for *E. histolytica*, a diagnosis of amebic pericarditis was made. Notably, the diagnosis was missed initially and was made retrospectively by performing PCR testing. The patient improved with metronidazole 750 mg thrice daily for 14 days, followed by paromomycin 500 mg thrice daily for 10 days.

**Conclusions:**

This case suggests that, although only 122 cases of amebic pericarditis have been reported, clinicians should be aware of *E. histolytica* as a potential causative pathogen. The polymerase chain reaction method was used to detect *E. histolytica* in the pericardial effusion and was found to be useful for the diagnosis of amebic pericarditis in addition to the positive results for the serum antibody testing for *E. histolytica*. Because of the high mortality associated with delayed treatment, prompt diagnosis should be made.

## Background

Amebic pericarditis was first reported by Rouis in 1860 during an autopsy of a patient with an hepatic amebiasis [1]. The three stages of amebic pericarditis are (1) “pre-suppurative pericarditis,” an initial sympathetic or reactive effusion, (2) “suppurative pericarditis,” a rupture of a liver lesion into the pericardial cavity with the characteristic anchovy paste appearance, often resulting in cardiac tamponade, and (3) “constrictive pericarditis,” a late complication of amebic pericarditis which develops over weeks to months and requires pericardiectomy [2, 3]. The pre-suppurative form should be considered a potential precursor of the suppurative form. Amebic pericardial effusion usually results from the extension of a left lobe liver lesion through the diaphragm into the pericardium, leading to a hepatopericardial fistula [4, 5]. Hematogenous dissemination can also occur, although less frequently.

*Entamoeba histolytica* (*E. histolytica*) is rarely identified as a cause of pericarditis. We report a case of amebic pericarditis in which the cause of the cardiac tamponade was not identified initially, and the diagnosis was made retrospectively when the patient deteriorated 1 month later, by performing PCR on a stored sample of pericardial fluid obtained during the first admission.

## Case presentation

A 71-year-old man without a history of travel to amebiasis endemic areas presented to our emergency department with a 2-week history of left pleuritic chest pain and a non-productive cough. The patient had a recent past medical history of essential thrombocytopenia and myelodysplastic syndrome/myeloproliferative neoplasm was treated with hydroxyurea (500 mg) followed by anagrelide hydrochloride hydrate (3 mg). Chest radiograph revealed cardiomegaly, pulmonary congestion, and left pleural effusion. The transthoracic echocardiogram (TTE) showed normal left ventricular wall motion with a small amount of pericardial effusion near the left ventricular posterior wall. The patient was admitted to the cardiology department on suspicion of anagrelide-induced acute heart failure; thus, treatment with anagrelide was stopped. The hospital course was favorable until hospital day 9 when the patient developed chest pain and dyspnea. The patient was in severe distress and had a blood pressure of 80/66 mmHg, heart rate of 104 beats per minute, respiratory rate of 24 breaths per minute, and an oxygen saturation of 77% on room air. Electrocardiogram showed ST-segment elevation in V2–6 and PR-segment depression in V3–6. TTE and computed tomography (CT) without contrast showed the increased pericardial fluid effusion. The patient was diagnosed with pericardial tamponade and required an urgent pericardiocentesis. The pericardial fluid was blood-colored with cell count of 4250 cells/µL, predominantly neutrophils (88%) with lymphocytes (6.5%), and eosinophils (0.5%). Many clusters of dead or degenerated cells were observed in cytology. We did not identify amebic trophozoites. The bacterial and mycobacterial cultures of the pericardial fluid were negative, as were serum IgM antibodies for viruses including *Echovirus*, *Coxsackievirus*, and *Parvovirus* B19. Rheumatoid and antinuclear antibodies were also negative. Although the cause could not be identified, chest pain and cough gradually improved with ibuprofen and colchicine. The patient was discharged on hospital day 13 after the pericardial effusion had resolved. However, 1 month after discharge and 5 days before the second admission, the patient presented with acute onset of fever, right upper abdominal pain, nausea, and loss of appetite. TTE revealed small amount of pericardial effusion, and contrast-enhanced CT scan of the abdomen revealed multiple liver lesions.

We empirically started with intravenous (IV) ceftriaxone (2 g every 24 h) and metronidazole (1 g every 8 h). Percutaneous drainage of the liver lesion was performed on hospital day 2. The liver lesion was neutrophil-dominant, with erythrocytes, lymphocytes, and histiocytes visible. Many clusters of dead or degenerated cells were observed on cytology as well as in the pericardial fluid. After drainage, the patient’s signs and symptoms gradually improved. Notably, on hospital day 12, serum enzyme immunoassay (EIA) was positive for *E. histolytica* IgM and IgG antibodies. Moreover, we performed conventional qualitative polymerase chain reaction (PCR) testing of DNA samples extracted from tissue samples of the liver lesion, and pericardial and pleural effusion fluid collected and stored during the first admission, using a dysentery amoeba-specific primer that partially amplified the 18S ribosomal DNA region. The PCR test result was positive. We diagnosed an amebic liver lesion associated with amebic pericarditis. We reported the positive PCR result to the pathologists and asked them to check again, but unfortunately, they could not confirm free living ameba. Of note, the patient initially denied any recent sexual history, but when we interviewed him again after he was diagnosed with amebiasis, he admitted that he had had sexual intercourse with several commercial sex workers 4 months previously. He denied any sexual contact with men, but he stated he had oral-anal sex. The dose of metronidazole was increased (to 750 mg IV every 8 h) and continued for 14 days, followed by oral paromomycin (500 mg every 8 h) for 10 days. The patient was clinically stable and was discharged on day 18 of hospitalization. Follow-up TTE and CT abdomen revealed no evidence of recurrence at the 1-year follow-up.

### Systematic review

**Fig. 1:**
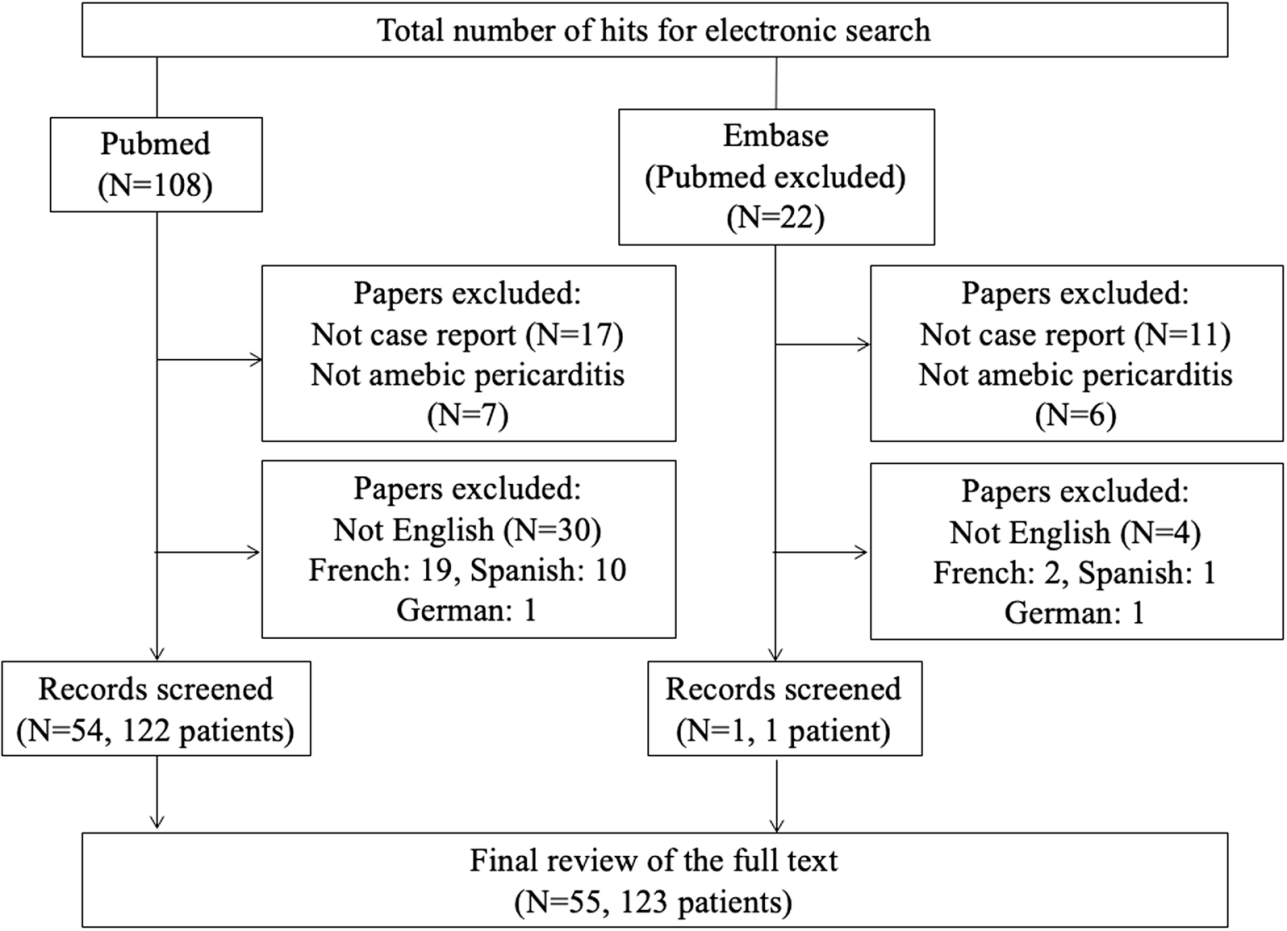
A flow chart of the systematic review data collected for this study

A literature search of the PubMed database (up to November 2019, updated in April 2021) was conducted using the keywords (((“Amoeba“[Mesh] OR amoeba[TIAB] OR ameba[TIAB] OR amebic[TIAB]) OR (“Amebiasis“[Mesh] OR amebias*[TIAB] OR amoebias*[TIAB])) AND (“Pericarditis“[Mesh] OR pericarditis[TIAB])) OR ((“Heart Diseases/parasitology“[Mesh]) AND ((“Amoeba“[Mesh] OR amoeba[TIAB] OR ameba[TIAB] OR amebic[TIAB]) OR (“Amebiasis“[Mesh] OR amebias*[TIAB] OR amoebias*[TIAB]))). The Embase database was also searched using the keywords (((‘amoeba’/exp OR amoeba) OR ameba OR amebic OR ‘amebiasis’ OR amebias* OR amoebias*) AND ‘pericarditis’) NOT (((‘amoeba’/exp OR amoeba) OR ameba OR amebic OR ‘amebiasis’ OR amebias* OR amoebias*) AND ‘pericarditis’ AND ([medline]/lim OR [pubmed-not-medline]/lim)) (Fig. 1). The database including titles, abstracts, and languages was firstly created by FK using the abovementioned formula. For the literature review, the database records were split in half (A and B). Database A was independently reviewed by KI and RH and database B was independently reviewed by TS and TF. Full-text text was used for eligibility assessment, and detailed information on each case was extracted. Finally, each database was checked by KK and TM to create the combined list of cases. Case reports that were not regarding amoebic pericarditis, and reviews and clinical studies that did not present cases of amoebic pericarditis were excluded. Additionally, reports for which abstracts or full texts were in languages other than English were excluded.

We found 53 papers describing 121 cases of pericarditis caused by *E. histolytica* [1, 3–55]. The detailed information on the clinical characteristics of these 122 cases, which includes our case, are shown in Table 1. Of those cases, 90.4% were male, the median age was 46 years, and only six cases, including this case, were reported after 2000. Moreover, 45.1% (n = 55) of the cases were reported from Asia, followed by Africa (n = 34, 27.9%), Central and South America (n = 11, 9.0%), North America (n = 6, 4.9%), and Europe (n = 3, 2.5%). Liver lesions were noted in 75% (n = 60) of cases, mostly in the left lobe (n = 32, 53.3%), followed by location data not available (n = 20, 33.3%), the right lobe (n = 7, 11.7%), and both lobes (n = 1, 1.7%). Six cases (2.4%) did not have a liver lesion. The median duration of antimicrobial therapy was 14 days, and 31.1% (n = 38) were fatal.

**Table 1:** Characteristics of patients with amebic pericarditis

No.	Case reference	Age	Sex	Year	Country	Chief complaint	Underlying diseases	Travel history	Liver abscess	Diagnosis	Biopsy findings	Surgical intervention	Antimicrobial used	Outcome	Follow up
1	Laha [6]	17	F	1946	India	Fever for 2 days	N/A	N/A	Yes, R	The response to treatment	N/A	Pericardiocentesis	Emetine 3 g	Cure	N/A
2	Berberian [7]	59	M	1949	The United States	Malaise, weakness, diarrhea	N/A	N/A	No	Biopsy of the wall of the umbilical sinus was positive for *E. hystolytica*	N/A	N/A	Aureomycin	Died	N/A
3	Beemer [8]	27	M	1956	Canada	Left sided chest pain	N/A	N/A	Yes, L	Autopsy	N/A	N/A	Streptomycin, isoniazid	Died	N/A
4	Downie [9]	2	M	1960	Nigeria	Cough, discharging ears, vomiting and diarrhea	None	None	Yes, L	Amoeba was seen in the connective tissue outside the pericardium.	The pericardial surface of the heart was covered by thick fibrin	N/A	N/A	Died	N/A
5	Paulley [5]	51	M	1965	United Kingdom	Burning precordial pain	Ischemic infarction	Residence in India 20 years before admission	Yes, L	The response to treatment	N/A	None	Penicillin, chloramphenicol, chloroquine, emetine	Cure	N/A
6	Mullan [10]	73	M	1965	United Kingdom	Upper abdominal pain, anorexia, hemoptysis over 1 month	N/A	Residence in Mesopotamia 50 years before admission	Yes, R	The presence of confirmed amebic liver abscess and the response to treatment	None (Liver: Actively motile amoebae,containing red cells, were present)	Pericardiocentesis	Emetine	Died	None
7	Gelfand [11]	12	M	1966	Zimbabwe	Right chest pain for 3 weeks, shortness of breath, weight loss, and fever	None	None	Yes,	Bloodstained fluid, and the response to treatment	Dense fibrous tissue with a sparse chronic inflammatory cellular infiltrate and with a small part of its surface covered by a narrow zone of granulation tissue	Pericardiocentesis	Emetine	Cure	N/A
8	Kulpati [12]	23	M	1966	India	Prexia, upper abdominal pain, constipation	N/A	N/A	Yes	Liver aspiration contained trophozoites of *E. histolytica*	N/A	N/A	Yes, but not mentioned in detail	Cure	No recurrence 4 month after discharge
9	D’cruz [13]	21	M	1967	India	Severe retrosternal pain, vomited and fainted	N/A	N/A	Yes	Pericardial fluid contained trophozoites of *E. histolytica*	N/A	None	No	Died	N/A
10	Rab [14]	49	M	1967	Pakistan	Retrosternal pain, dyspnea	N/A	N/A	Yes, L	Anchovy sauce pus and the response to treatment	Fibrinous deposit	Pericardiocentesis	N/A	Died	None
11	Cook [15]	25	M	1970	Malaysia	Fever, cough, bloody sputum	None	N/A	Yes, R	The response toemetine and a strongly positive amebic fluorescent antibody test	None	Pericardiocentesis, laparotomy of left subphrenic abscess	Emetine and chloroquine	Cure	N/A
12	Ganeshananthan [16]	36	M	1971	Sri Lanka	Dry cough and chest pain for 3 weeks	N/A	N/A	N/A	Thick brown pus, having the consistency of typical amoebic liver abscess pus	N/A	Pericardiocentesis	Emetine, metronidazole, chloroquine	Cure	No recurrence 3 years after discharge
13	Bansal [17]	19	M	1971	India	Fever, dry cough, right shoulder pain for 5 days	N/A	N/A	Yes, R	Anchovy sauce pus and the response to treatment	None	Pericardiocentesis	Emetine, chloroquine, di-iodohydroxy quinoline	Cure	No recurrence 16 months after discharge
14	Kapoor [18]	27	M	1972	India	High fever with pain in the left chest and subcostal region	Gastro-pericardial fistula	N/A	Yes, L	N/A (Stool: trophozoites of *E. histolytica*)	N/A	Pericardiocentesis	Intramuscular emetine	Cure	NA
15	Kapoor [18]	30	M	1972	India	General weakness, pain in the abdomen and swelling in the epigastrium for 3 weeks	N/A	N/A	Yes, L	N/A (Stool: trophozoites of *E. histolytica*)	N/A	Pericardiocentesis	Emetine, chloroquine	Cure	N/A
16	Kapoor [18]	34	M	1972	India	Pain in the epigastrium and right lumber region for 2 months	N/A	N/A	Yes	Anchovy sauce pus from the front of pericardial cavity	N/A	Pericardiocentesis, thoracotomy	Emetine	Cured	N/A
17	Kapoor [18]	35	M	1972	India	Sudden onset of severe generalized chest pain and dyspnea for 12 h	N/A	N/A	Yes, L	N/A (Liver: trophozoites of *E. hystolytica*)	Yes	Pericardiocentesis	N/A	Died	N/A
18	Kapoor [18]	25	M	1972	India	Pyrexia for 5 days	N/A	N/A	N/A	N/A	N/A	Pericardiocentesis	Dehydroemetine, chloroquine	Cured	No recurrence 3 weeks after discharge
19	Kapoor [18]	50	M	1972	India	Fever for 2 months and pain in the right hypochonrium	N/A	N/A	Yes, L	Anchovy sauce pus from the pericardial cavity	N/A	Pericardiocentesis	Emetine, chloroquine	Cured	No recurrence two years after discharge
20	Spiegel [19]	48	M	1972	The United States	Abdominal pain of right upper quadrant	N/A	N/A	Yes, R	Pericardial fluid contained trophozoites of *E. histolytica*	N/A	N/A	Metronidazole and chloroquine	Cure	N/A
21	Heller [20]	42	F	1972	United States	Dyspnea, weight loss, left upper abdominal pain for 8 days	N/A	Move from Mississippi to Chicago when she was 3 years old	Yes, L	Anchovy sauce pus, the response to treatment, and indirect hemagglutination test (1:2560)	Fibrinous pericarditis	Pericardiocentesis followed by pericardiectomy	Metronidazole 750 mg thrice daily for 3 weeks	Cure	N/A
22	Heller [20]	47	M	1972	United States	Retrosternal pain, sweating, dyspnea for 1 day	N/A	Move from Mexico to Chicago 5 months previously	Yes, L	Anchovy sauce pus, the response to treatment, and indirect hemagglutination test (1:5128)	Fibrinous pericarditis	Pericardiectomy	Emetine (next day changed to dehydroemetine), chloroquine	Died	None
23	Anderson [21]	N/A	M	1972	United States	Left pleuritic chest pain, jaundice for 10 days	None	N/A	Yes, L	Anchovy sauce pus and the response to treatment	None	Pericardiocentesis followed by pericardiectomy	N/A	Cure	N/A
24	Suryanarayan [22]	40	M	1973	India	Pain in the epigasric region and hypochondrium for one mouth, and dysponea for 1 week	N/A	N/A	Yes, L	N/A	N/A	Pericardiocentesis	Dehydroemetine, tetracyclines, chloroquine and didoquin	Cure	N/A
25	Suryanarayan [22]	45	M	1973	India	Fever for 3 months and dyspnea for 2 days	N/A	N/A	Yes, L	Pericardial fluid contained trophozoites of *E. histolytica*	N/A	None	N/A	Died	N/A
26	Lewis [23]	35	M	1973	N/A	N/A	N/A	N/A	N/A	N/A	N/A	N/A	N/A	N/A	N/A
27	Ganeshananthan [16]	33	M	1974	Sri Lanka	Left-sided chest pain and shortness of breath for 2 weeks	N/A	N/A	N/A	The fluorescent antibody test was positive	N/A	Pericardiocentesis	Emetine, metronidazole	Cure	No recurrence 6 months after discharge
28	Ganeshananthan [16]	43	M	1974	Sri Lanka	Shortness of breath and pain in the chest and abdomen for 18 days	N/A	N/A	Yes	Typical amoebic pus was drawn out from needling of the pericardium	N/A	Pericardiocentesis	Emetine, tetracycline, metronidazole	Cure	N/A
29	Ganeshananthan [16]	32	N/A	1974	Sri Lanka	Pain in the left upper abdomen for 2 months	N/A	N/A	N/A	The fluorescent antibody test was positive	N/A	Pericardiocentesis	Emetine and chloroquine followed by metronidazole	Cure	N/A
30	Guimaraes [24]	33	M	1974	Brazil	Fever, chills, dry cough, retrosternal pain, weight loss, dyspnea, diarrhea	N/A	N/A	Yes, L	Anchovy sauce pus and the response to treatment	N/A	Pericardiocentesis	Chloroquine 2 g daily for 7 days, followed by 500 mg daily for 23 days, metronidazole 2 g daily for 30 days, emetine hydroxychloroquine 60 mg daily for 10 days plus the injection of 75% hypaque into the pericardial cavity	Cure	N/A
31	Faerber [25]	35	M	1974	South Africa	Pleuritic chest pain, productive cough, hemoptysis for 2 months	N/A	N/A	Yes, R and L	N/A	N/A	N/A	N/A	Cure	N/A
32	Haranath [26]	55	M	1974	India	Exertional dyspnea, distention of the abdomen and edema of the left upper limb for 10 days	Rheumatic fever	N/A	N/A	Anchovy sauce pus from the cardiac cavity	N/A	Pericardiocentesis	Dehydroemetine, metronidazole	Cure	NA
33	Ganesan [3]	40	M	1975	India	Progressive general weakness, chest pain, dyspnea for 20 days	N/A	N/A	Yes, L	Anchovy sauce pus and the response to treatment	None	Pericardiocentesis	Dehydromethine 30 mg daily for 10 days, chloroquine 250 mg thrice daily for 10 days, and metronidazole 1200 mg daily for ten days	Cure	N/A
34	Agrawal [1]	40	M	1975	India	Fever, cough, pain in right hypochondrium, fatigue for 2 months	None	N/A	Yes, L	Aspiration of anchovy source pus from the cardiac cavity	The nonspecific chronic inflammatory reaction of the pericardium	Pericardiocentesis	Metronidazole 400 mg thrice daily	Died	None
35	Agrawal [1]	25	N/A	1975	India	Pain in the upper abdomen and dry cough for 2 weeks	None	N/A	Yes, L	Aspiration of anchovy source pus from the cardiac cavity	An actively inflamed fibrosing pyopericardium of nonspecific nature	Pericardiocentesis	Metronidazole 400 mg thrice daily	Cure	No recurrence 6 months after discharge
36	Kulpati [27]	34	M	1976	India	Moderately high intermittent fever and dull aching pain in right hypochondrium for 1 month	History of amoebic dysentery about 8 months back	N/A	Yes, L	The pericardial fluid was chocolate colored. The direct smear and culture of pericardial fluid demonstrated *E. hystolytica*	N/A	Pericardiocentesis	Metronidazole and emetine	Died	N/A
37–63	Adams EB1-27 [28﻿]	N/A	N/A	1977	South Africa	N/A	N/A	N/A	8 patients (mostly L)	N/A	N/A	N/A	N/A	Cure (n = 19) Died (n = 8)	N/A
64	Muralidar [29]	19	F	1977	India	Pyrexia and pain in the right upper abodomen for 2 weeks and breathlessness for 4 days	None	N/A	N/A	Anchovy sauce pus from the cardiac cavity	N/A	Pericardiocentesis	Emetine, metronidazole, and tetracycline	Died	N/A
65	Muralidar [29]	52	M	1977	India	Chest pain and breathlessness since 3 days	History of amoebic dysentery	N/A	N/A	Anchovy sauce pus from the cardiac cavity, serum indirect hemagglutination	N/A	Pericardiocentesis	Emetine and metronidazole	Cure	No recurrence 6 weeks after discharge
66	Kundu [30]	25	F	1978	India	Pain in right hypochondrium associated with moderately high intermittent fever for 15 days and difficulty in breathing for 1 week	None	N/A	Yes, L	Serum gel diffusion test, chocolate pus	N/A	Pericardiocentesis	Metronidazole followed by chloroquine	Cure	No recurrence 4.5 months after discharge
67	Bellosillo [31]	37	M	1978	Philippine	Shortness of breath	N/A	N/A	Yes	Pericardial fluid contained trophozoites of *E. histolytica*	N/A	Pericardiocentesis	Tinidazole	Cure	No recurrence 3 months after discharge
68–72	Verghese 1-5 [32]	N/A	N/A	1979	India	N/A	N/A	N/A	N/A	N/A	N/A	Pericardiocentesis (n = 1), pericardiotomy (n = 3)	N/A	Cure (n = 3) Died (n = 2)	N/A
73	Almeida [33]	35	M	1979	India	Epigastric pain for 1 month and swelling in upper abdomen for 15 days	None	N/A	Yes, L	Anchovy sauce pus and the response to treatment	N/A	Pericardiocentesis	Emetine and chloroquine	Cure	No recurrence 9 months after discharge
74	Almeida [33]	20	M	1979	India	Shortness of breath and left chest pain for 3 days	None	N/A	Yes, L	Anchovy sauce pus from the cardiac cavity	Pericardium was thickened	Pericardiocentesis	Emetine, and oxytetracycline	Died	N/A
75	Almeida [33]	25	M	1979	India	Left chest pain, cough, and fever for 1 month	None	N/A	Yes, L	Anchovy sauce pus from the cardiac cavity	N/A	Pericardiocentesis	N/A	Died	N/A
76	Almeida [33]	40	M	1979	India	Anorexia, fever, and right chest pain for 15 days	None	N/A	Yes, L	N/A	Autopsy revealed liver abscess with pericarditis and cardiac tamponade	None	N/A	Died	N/A
77	Almeida [33]	50	F	1979	India	Jaundice and fever for 1 month	None	N/A	Yes, L	Bloodstained fluid	N/A	Pericardiotomy	Emetine and metronidazole	Died	N/A
78–89	Tyagi 1–12 [34]	4–55	M:9 F:3	1980	India	Fever (n = 10), dyspnea (n = 5), cough (n = 4), diarrhea (n = 3	N/A	N/A	Yes, L: 5, R: 4	Anchovy sauce pus from the cardiac cavity (n = 2), pericardial fluid contained trophozoites of *E. histolytica* (n = 2)	N/A	Pericardiocentesis	N/A	Cure (n = 4) Died (n = 8)	N/A
90	Gupta [35]	43	M	1980	India	Dyspnea, chest pain	N/A	N/A	No	Pericardial fluid contained trophozoites of *E. histolytica*Autopsy	N/A	Pericardiocentesis	N/A	Cure	N/A
91	Kala [36]	35	M	1980	India	Dyspnea, diarrhea	N/A	N/A	Yes	Pericardial fluid contained trophozoites of *E. histolytica*Autopsy	N/A	Pericardiocentesis followed by pericardiectomy	Emetine, Streptomycin, isoniazid	Cure	N/A
92–101	Ibarra-Perez 1–10 [37]	N/A	N/A	1981	Mexico	N/A	N/A	N/A	N/A	N/A	N/A	N/A	N/A	N/A	N/A
102	Adeyemo [38]	N/A	N/A	1984	Nigeria	N/A	N/A	N/A	Yes, L	N/A	N/A	Pericardiocentesis	N/A	Died	N/A
103	Adeyemo [38]	N/A	N/A	1984	Nigeria	N/A	N/A	N/A	Yes, L	N/A	N/A	Pericardiocentesis	N/A	Died	N/A
104	Farer [39]	23	M	1984	Vietnam	Dyspnea, chest pain	N/A	Indonesia	N/A	Microscopic finding of amebic trophozoites, anchovy sauce pus, and the response to treatment	None	Pericardiocentesis	Specific chemotherapy for amebiasis	Cure	N/A
105	Shanker [40]	30	M	1985	India	Irregular fever and dull aching oain in right hypochondrium for 1 month	History of amoebic dysentery about 6 months back	N/A	N/A	Pericardial fluid contained trophozoites of *E. histolytica*Autopsy	N/A	Pericardiocentesis	Metronidazole, ampicillin, emetine hydrochloride	Cure	N/A
106	Pirie [41]	N/A	N/A	1986	South Africa	N/A	N/A	N/A	N/A	N/A	N/A	N/A	N/A	N/A	N/A
107	Baid [42]	48	M	1987	India	Dyspnea, left chest pain, fever, edema of the feet, weakness for 1 month	N/A	N/A	Yes, R	Anchovy sauce pus and the response to treatment	Subepicardial fat-containing proliferating fibroblasts and lymphocytic infiltrate	Pericardiocentesis followed by pericardiectomy	Metronidazole	Cure	None
108	Strang [43]	N/A	N/A	1987	South Africa	N/A	N/A	N/A	Yes	N/A	N/A	N/A	N/A	N/A	N/A
109	Blackett [44]	50	M	1988	Cameroon	Left-sided chest pain, dyspnea, abdominal distension, edema for several days	N/A	N/A	None	The positive serological tests (1/1024) for amoebiasis, the appearance of the fluid obtained on tapping the pericardium, and the response to treatment	None	None	Metronidazole and chloroquine	Died	None
110	Vanachayangkul [45]	51	M	1989	Thailand	Epigastric oppression and fever for 1 week beginning with severe pain, occasionally colicky in nature without nausea or vomiting	N/A	N/A	Yes	Aspiration of pericardial effusion yielded 350 mL of brownish grey pus with numerous trophozoites of active motile *E. hystolytica*	N/A	Pericardiocentesis followed by pericardiotomy	Metronidazole and cefotiam and amikacin	Cure	N/A
111	Lami [46]	26	F	1989	Italy	Fever, headache, left chest pain	None	Tunisia for 2 months	None	Indirect hemagglutination test	N/A	N/A	Metronidazole 2 g daily for 10 days	Cure	N/A
112	Supe [47]	40	M	1991	India	N/A	N/A	N/A	Yes	Elisa test for *E. histolytica* antibody was positive	N/A	Pericardiocentesis	Emetine, metronidazole and amikacin	Cure	N/A
113	Gomersall [48]	28	M	1994	United Kingdom	Fever, night sweats, pyrexia, and right-sided pleuritic chest pain for 6 weeks	None	Stationed in Kenya	Yes, L	A fluorescent antibody test for *E. hystolytica* was positive (titer 1/320) and amoebic enzyme immunoassay was positive at 120 %	None	Pericardiocentesis	Metronidazole for 10 days and diloxanide for 10 days	Cure	No recurrence 6 months after discharge
114	Perna [49]	37	M	1994	Italy	Fever, malaise, weight loss	Drug addiction	N/A	Yes, L	A bacteriological specimen from the pericardial fluid yielded *E. hystolytica*, anchovy sauce pus, and the response to treatment	N/A	Pericardiectomy	Chloroquine	N/A	N/A
115	Shandera [50]	64	M	1998	India	Epigastric pain and pleuritic pain for 6 weeks	N/A	N/A	Yes, L	Positive serological tests for amoebiasis	None	Pericardiocentesis	Metronidazole and diiodoquinolol	Cure	N/A
116	Chao [4]	44	M	1998	Taiwan	Dyspnea for the previous 2 days, mucoid diarrhea for 3 days	Chronic schizophrenia	N/A	Yes, L	Amebic antibodies by hemagglutination test (1:16,384)	Fibrinous exudate with polymorphic neutrophilic cell infiltration	Pericardiectomy	Metronidazole 500 mg 4 times daily for 2 weeks	Cure	No recurrence 1-year after discharge
117	Wiwanitkit [51]	N/A	N/A	2008	Thailand	Chest pain, discomfort, and arrhythmia	N/A	N/A	Yes	N/A	N/A	N/A	N/A	Cure (n = 2) Died (n = 1)	N/A
118	Murali [52]	38	M	2011	India	Fever and right hypochondrial pain for 10 days	N/A	N/A	Yes, R	Positive linked immunosorbent assay for *E. hystolytica* (both IgM and IgG)	None	Pericardiocentesis	Meropenem, metronidazole	Cure	N/A
119	Amine [53]	38	M	2015	Morocco	Fever, shortness of breath, and leg swelling for 2 months	Recurrent diarrhea	N/A	None	Trophozoites of *E. hystolytica* (pleural effusion)	None	Pericardiectomy	Metronidazole 500 mg trice daily	N/A	N/A
120	Agarwal [54]	25	M	2019	India	Abdominal pain and fever for 1 month	None	N/A	Yes, L	Anchovy sauce pus, PCR for *E. hystolytica* from the pericardial cavity	N/A	Pericardiocentesis, pleural pericardial window	Metronidazole	Cure	No recurrence at 1 year after discharge
121	Keleş [55]	25	M	2020	India	Fever, chills, and pain in the upper abdomen for 1 month	N/A	N/A	Yes, L	Reddish-brown pus from the cardiac cavity, positive serological test for amebiasis, and PCR for *E. hystolytica*	N/A	Pericardiocentesis	Metronidazole followed by oral luminal amebicide diloxanide	Cure	No recurrence 1-year after discharge
122	Francis [56]	15	M	2020	Turkey	Diarrhea and abdominal pain for 2 days	None	N/A	None	*E. hystolytica* trophozoites and cysts in fresh stool, and the response to treatment	None	None	Metronidazole (30 mg/kg/day)	Cure	No recurrence 6-month after discharge
123	Raza [57]	7	M	2020	Pakistan	Fever and abdominal pain for 15 days and diarrhea for 3 days	Acute gastroenteritis	N/A	Yes, L	Cysts of *E. histolytica* and positive enzyme-linked immunosorbent assay in stool	None	None	Metronidazole for 14 days, followed by diloxanide furoate for the next 7 days	Cure	No recurrence 2-week after discharge
Current case	Matsuo^a^	71	M	2019	Japan	Epigastric pain, fatigue for 2 months	Essential thrombocytosis treated with anagrelide	Asia	None	Positive serological test for amebiasis, PCR for *E. hystolytica* from the pericardial cavity	N/A	Pericardiocentesis	Metronidazole 750 mg trice daily for 2 weeks, followed by paromomycin 500 mg trice daily for 10 days	Cure	No recurrence at 1 year after discharge

F, female; M, male; R, right; L, left; *E. hystolytica*, *Entamoeba histolytica* PCR, polymerase chain reaction; N/A, not available

^a^Authors of current case

## Discussion and conclusions

To the best of our knowledge, this is the first systematic review of amebic pericarditis as a rare complication of amebiasis. A strength of this review is that, in addition to providing patient demographics for 122 cases over the past 80 years, it also focuses on the use of PCR, which has recently been shown to be useful for diagnosis for amebiasis. The learning point from this case is that had the diagnosis had been made at the time of the first cardiac tamponade, the second hospitalization might have been avoided. The diagnosis was missed initially and confirmed retrospectively when the patient deteriorated 1 month later, by performing PCR on a stored sample of pericardial fluid obtained during the first admission. It is worth noting that although we did not suspect amebiasis because the patient initially denied a history of sexual intercourse and did not have a history of travel to an endemic area. We learned that if the cause of pericarditis is unclear, a detailed interview should be conducted and amebiasis should be suspected.

Most cases of amebic pericarditis were reported from developing countries, especially the tropics and subtropics in Asia and Africa, where there is inadequate hygiene and access to sanitation [58]. This trend is similar to that of all amebic dysentery and amebic liver lesion [58] The previous study in Egypt showed that approximately 40% of patients with acute diarrhea had amebic colitis [59]. The reason why the number of case reports peaked in the 1970s and has been gradually decreasing subsequently, may be because treatment has been standardized and is generally successful, so clinicians are less likely to report cases. In developing countries where amebiasis are endemic, it is still possible that this has not yet been reported due to underdevelopment of surveillance and diagnostic techniques [58]. Even not in endemic areas, as our case, sexual intercourse could be the risk factors of amebiasis.

Regarding the location of the liver lesion, in our literature review, we found that 32 cases (53.3%) had left lobe liver lesion, seven (11.7%) with only right lobe liver lesion, and six case (2.4%) without a liver lesion was observed. Although it has been known that amebic pericarditis is often associated with left-sided liver lesions, this result suggested that amebic pericarditis is not necessarily only secondary to the left lobe of the liver. The absence of an initial liver lesion in our case made the diagnosis of amebic pericarditis difficult, but this case taught us that PCR testing for amebiasis should be performed promptly when investigating cases of pericarditis of unknown origin. The fact that the pericarditis preceded the liver lesion in our case, and that the pericardial fluid was bloody, not like anchovy paste, suggests that the mechanism of spread to the pericardium was by hematogenous dissemination. Anchovy paste-like liquid is seen in cases of liver lesion, and consists of ameba bound to necrotic liver cells. In fact, considering that other extraintestinal amebiasis, such as central nervous system lesions and pulmonary amebiasis without liver lesions are assumed to be disseminated hematogenously [58, 60], trophozoites could have disseminated into the pericardial sac via the bloodstream.

In terms of diagnosis, anchovy paste-like fluid by pericardiocentesis was reported to be the main characteristic and was found in about half of the cases evaluated in this review. Since the 1970 s, there has been an increase in the number of cases in which a positive antibody test was combined with pericardiocentesis [3, 18]. Until then, most cases had been clinically diagnosed only by the characteristic pericardial fluid findings of “anchovy paste” and response to treatment. Considering that trophozoites could be seen microscopically in only two cases (7.1%), the absence of trophozoites could not rule out amebic pericarditis. Notably, the most recent reports revealed the usefulness of PCR detection of *E. histolytica* in pericardial fluid [49, 51]. Since PCR for *E. histolytica* has been reported to be useful in intestinal and extraintestinal amebiasis [61], clinicians should consider using PCR methods to confirm *E. histolytica* in patients with pericarditis of unknown origin.

The conventional qualitative PCR method used in this study has been shown to be highly specific and sensitive by testing with DNA from a variety of pathogens, including bacteria and other protozoa [62]. Unfortunately, we did not perform serum quantitative PCR in this case. It would have been informative to compare the results of serum quantitative PCR between first and second admissions. Compared with conventional PCR, real-time PCR has the following advantages: (1) the results are easier to interpret numerically than by visually checking the stained gels as in conventional PCR; (2) the sensitivity is higher; (3) no post-amplification analysis is required, minimizing the risk of contamination of the laboratory; (4) the duplex profile can distinguish between *E. histolytica* and *E. dispar* infections [63]. Nevertheless, real-time PCR is more costly than morphological stool examination and antigen-based detection tests. Therefore, real-time PCR is unaffordable in many of the countries in which *E. histolytica* is endemic. Instead, this technique may be useful in developed countries for diagnosing amebiasis in travelers from endemic areas.

Our literature review showed that, in most cases, pericardiocentesis was performed to investigate the cause of pericarditis. However, as in our case, it is essential to note that cardiac tamponade may occur, requiring urgent puncture or pericardiotomy. In this review, approximately 30% of patients died. In patients with tamponade early surgical intervention is essential, regardless of the cause. Regarding antimicrobial options, metronidazole was most commonly used, followed by emetine. The dosage and optimal duration of metronidazole varied widely. Still, as recommended for other amebic infections [58], most of the patients received 500–750 mg three times a day for 10–14 days (median = 14 days).

An important limitation of this study is that many of the cases were old and could not be directly compared due to differences in diagnostic tools. Due to the unavailability of accurate diagnostic tests, there are likely to have been many additional cases that were not diagnosed or reported. Furthermore, it should be noted as a limitation that manuscripts published in languages other than English are excluded in this review, as shown in Fig. 1. There may be differences in the characteristics of amoebiasis in countries where English is not the native language. Moreover, the recurrence rate or long-term prognosis could not be determined because of a lack of information. Further cases should be accumulated, and research on the appropriate diagnosis and management of amebic pericarditis is warranted.

In summary, although amebic pericarditis is rare, it should be promptly diagnosed and treated because of its high mortality rate. In addition to serum antibodies, PCR obtained by pericardiocentesis should be considered for patients with pericarditis of unknown origin and high pretest probability. It is also important for clinicians to be aware that pericarditis can be associated not only with the typical left lobe, but also with the right lobe, in the absence of liver lesion, or with delayed appearance, as in our case. This study highlights amebic pericarditis and contributes to the knowledge of its diagnosis.

## Data Availability

Not applicable.

## Abbreviations

CK: Creatine kinase
CT: Computed tomography
*E. histolytica*: *Entamoeba histolytica*
IV: Intravenous
PCR: Polymerase chain reaction
TTE: Transthoracic echocardiogram
